# Development of the scoliosis research society spinal deformity surgery safety checklist

**DOI:** 10.1007/s43390-024-00973-1

**Published:** 2024-10-23

**Authors:** Rafael De la Garza Ramos, Justin K. Scheer, Nabil Matmati, Lloyd A. Hey, Douglas C. Burton, Marinus de Kleuver, Christopher P. Ames, Vijay Yanamadala

**Affiliations:** 1 Department of Neurological Surgery, Montefiore Medical Center and Albert Einstein College of Medicine, New York, NY USA; 2 Department of Neurological Surgery, University of California San Francisco, San Francisco, CA USA; 3 Department of Neurosurgery, Hartford HealthCare and Quinnipiac University Frank H. Netter School of Medicine, Westport, CT USA; 4 Hey Clinic for Scoliosis and Spine Care, Duke Raleigh Hospital Campus, 3320 Wake Forest Road, Raleigh, NC 27609 USA; 5 Department of Orthopedic Surgery, University of Kansas Medical Center, Kansas City, KS USA; 6 Department of Orthopedic Surgery, Radboud University Medical Center, Nijmegen, The Netherlands

**Keywords:** Patient safety, Scoliosis surgery, Checklist, Adolescent idiopathic scoliosis (AIS), Adult spinal deformity (ASD)

## Abstract

**Purpose:**

Spine deformity surgery is a complex multi-step procedure that has a relatively high complication rate. The use of surgical safety checklists has been shown to reduce perioperative adverse events, but existing lists are varied and non-specific for spinal deformity surgery. Thus, the purpose of this study was to develop a comprehensive surgical checklist for complex spinal corrective surgery.

**Methods:**

An electronic survey consisting of 187 surgical checklist items that had been developed and used by a group of SRS members over a 5-year period was distributed to the Scoliosis Research Society Safety and Value Committee membership. The survey sections included: (1) pre-operative area, (2) initial operating room visit, (3) before turning, (4) positioning, (5) prepare and drape, (6) pre-incision timeout, (7) intraoperative, (8) finishing implant placement and confirming imaging, (9) final rods and locking, (10) prior to closure, (11) closure, (12) turn to supine, and (13) checkout/debriefing. Respondents graded each item on a five-point Likert scale based on their perceived importance and feasibility for inclusion in the checklist. Features graded as “moderately important” or “very important” to include by at least 70% of respondents were considered to meet the cutoff for inclusion-based standard Delphi practices. Study data were collated using REDCap.

**Results:**

A total of 25 surgeons completed the survey in its entirety. The overall checklist “package” was shortened to 9 individual checklist modules, with 2 to 16 items per checklist. In terms of individual checklist items, 40% of items (74 of 187) met the cutoff for inclusion; 17 of these items were graded as “very important,” which included verifying the presence of implantable devices, reviewing the surgical plan and positioning with the surgical staff, securing the endotracheal tube, bite block confirmation, prone and lateral positioning, neuromonitoring baseline readings, double-checking that the implant screw caps were locked prior to closure, and confirming that the patient was moving bilateral lower extremities before leaving the operating room when possible.

**Conclusion:**

This study has led to the development of a specific spinal deformity surgical checklist of 74 (many specific to spine surgery) items that were considered important for inclusion; 17 were considered “very important”.

**Supplementary Information:**

The online version contains supplementary material available at 10.1007/s43390-024-00973-1.

## Introduction

Corrective surgery for pediatric, adolescent, and adult spinal deformity (ASD) can provide significant improvement and maintenance of quality of life, posture and appearance, and prevention of future curve progression [1]. Nonetheless, these surgeries are complex and long multi-step procedures in a high-risk anatomic location due to the proximity of the spinal cord and major blood vessels and they can have a high complication rate. In ASD, for example, perioperative complication rates have been estimated at 55% for any complication and 18.5% for major adverse events [2]. While adolescent idiopathic scoliosis (AIS) is well known for having far fewer complications than ASD, the procedure itself is largely the same as ASD surgery, and many of the same risks, though less frequent still apply [3, 4]. Although complication avoidance can be challenging, data suggest that at least half of surgical complications are potentially avoidable [5, 6].

In 2009, the World Health Organization developed guidelines identifying practices that could ensure surgical safety worldwide [7]. Haynes et al. conducted an international multi-center study and found that the implementation of a surgical checklist based on the previously reported WHO guidelines resulted in significant reductions in perioperative morbidity and mortality [8]. In regards to reconstructive surgery for spinal deformity, experts advocate for standardization of the work process, all the way from preoperative optimization and surgical planning to intraoperative blood loss management, responses to neuromonitoring signal changes, and postoperative recovery [9]. This philosophy of work allows identification of work practices that produce value versus those that do not, specifically by eliminating ambiguity, variability, and unpredictability from the process [9]. For changes in neuromonitoring signals, for example, Vitale et al. published an evidence-based checklist that provides surgeons and the entire treatment team (anesthesiologists, neuromonitoring specialists, and nurses included) with a to-do list on how to respond to avoid permanent deficits [10]. Such checklists have been shown to help professionals perform complex tasks under pressure [9, 11].

Although surgical checklists are now implemented at most surgical centers, there is currently no specific checklist for spinal deformity surgery. Given that these procedures are complex, and can be performed on patients with comorbidities, it is imperative to optimize surgical safety. In this study, we surveyed surgeons from the Scoliosis Research Society (SRS) Safety and Value Committee and leadership of the SRS Research Council to identify important items to include in a surgical checklist specific for both pediatric and adult patients undergoing corrective spinal deformity surgery. The aim was to provide a comprehensive perioperative spinal deformity surgery safety checklist, for the whole surgical episode from preoperative holding to discharge from the operating room.

## Materials and methods

### Background

The vision to implement checklists in deformity surgery started in 2016 with a small group of SRS members interested in quality and safety improvement. In 2018, this group conducted a multi-site pilot study which found that it was feasible and beneficial to implement electronic checklists in the operating room to conduct scoliosis surgery [12]. Surgeons were free to create and share their checklists with other surgeons, and evolve their checklists time, working with members of their surgical teams and colleagues. In 2019, after 3 years of pilot work, a group of SRS Safety and Value Committee members agreed that it would be helpful to have a common series of surgical checklists to provide as a good base starting point for surgeons and their teams. Content from the existing checklists was again shared among the members, and a prototype series of 13 sections with 187 total checklist items was created. The list of items was gathered through expert opinion from SRS members (including senior author LAH) and previous experience with adverse events. This common spinal deformity surgery series of checklists was then used at pilot sites and found to be feasible and helpful for the operating room team, for pediatric, adolescent, and adult spinal deformity surgery.

In 2020, the SRS Safety and Value Committee was charged by SRS leadership with the development of a more concise version of the checklists, to enhance widespread adoption by spine surgeons and their teams around the globe. Each surgical team could then use that concise series of checklists as a starting point for their own quality and safety improvement efforts.

### Study design

An electronic survey consisting of the pilot 13 sections and 187 items to potentially be included in the surgical checklist was distributed to the SRS Safety and Value Committee between May 15 and July 10, 2021. The survey sections included: (1) Pre-Operative Area (14 items); (2) Initial Operating Room Visit (21 items); (3) Before Turning (17 items; (4) Positioning (20 items); (5) Prep and Drape (15 items); (6) Pre-Incision Timeout (34 items); (7) Intraoperative (18 items); (8) Finishing Screws and Confirming Imaging (5 items); (9) Final Rods and Locking (5 items); (10) Prior to Closure (7 items); (11) Closure (11 items); (12) Turn to Supine (11 items); and (13) Checkout/Debriefing (9 items).

Respondents graded each item on a five-point Likert scale based on its perceived importance for inclusion in the checklist: (1) not important at all, do not include; (2) not that important; (3) neither important nor unimportant; (4) moderately important, and (5) very important, must be included.

### Statistical analysis

Respondent data were collated and managed using REDCap electronic data capture tools hosted at Hartford HealthCare, Hartford, CT. An exploratory data analysis was performed, and responses were collected and described as percentages. All questionnaire items that were graded as “moderately important” or “very important, must be included” by at least 70% of respondents were considered to meet the cutoff for inclusion in the surgical checklist based on standard Delphi practices. Data were analyzed in Microsoft Excel (Microsoft, Redmond, Washington).

## Results

**Fig. 1:**
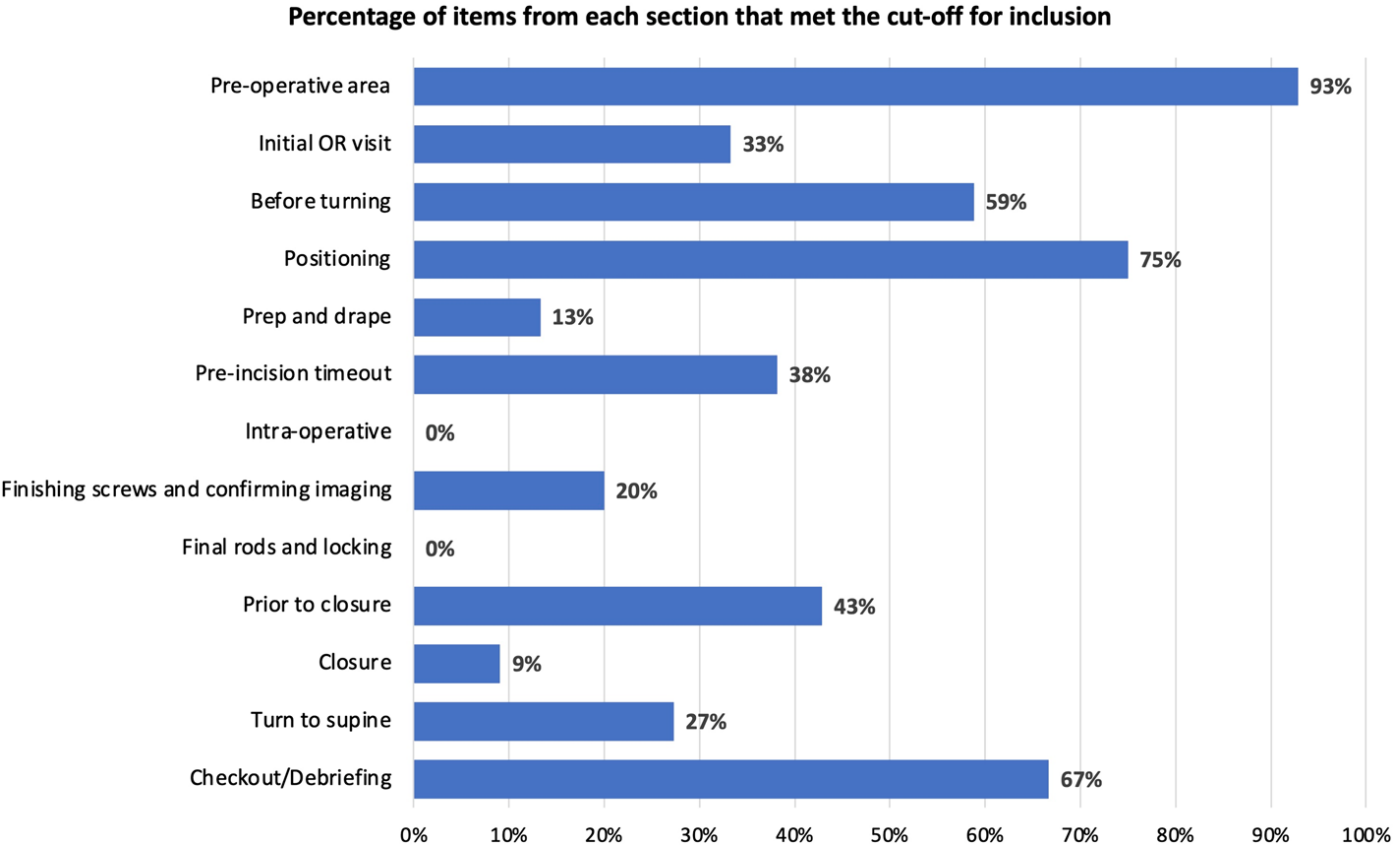
Percentage of items per section that met the 70% threshold for inclusion in the checklist

A total of 36 surgeons responded to the survey and 69% completed it in its entirety (25 of 36). Out of 187 items in the survey, 40% met the cutoff for inclusion in the surgical checklist (74 of 187) (Table 1). Of these selected 74 items, 23% (17 of 74) were considered “very important, must be included”. The proportion of items for each section that met the cutoff for inclusion in the checklist is shown in Fig. 1. The Pre-Operative Area section had 93% (13 of 14) of its items deemed “moderately or very important” to include in the checklist; this was followed by the Positioning section with 75% (15 of 20) and the Checkout/Debriefing section with 67% (6 of 9). None of the items in the Intraoperative or Final Rods and Locking sections met the cutoff for inclusion in the surgical checklist.

**Table 1:** Items in the checklist and percentage of surgeons who chose items to be “moderately” or “very important” to include

Section	# (*n* = 187)	Item	Percentage of surgeons who chose moderately or very important to include
Pre-Operative Area	1	No skin infections. No respiratory symptoms	85%**
	2	Confirm postop bed availability (ICU, PICU)	80%*
	3	Surgical and Blood consent signed and dated within hospital time window	78%**
	4	All jewelry and piercings removed	72%*
	5	Presence of any implantable devices (pacemaker, stimulators, pumps, joints) and safety with neuromonitoring and electrocautery	89%*
	6	Surgical site(s) marked with initials	84%*
	7	Chlorohexidine (CHG) wipe to surgical site	83%*
	8	Review TXA contraindication: clotting, stroke	79%*
	9	Allergies, and latex allergy?	88%**
	10	Nasal decolonization with 10% iodine swabs	45%
	11	Surgical and post-surgical plan reviewed with patient/family	76%*
	12	Patient and family questions answered	80%*
	13	Family member mobile number and name recorded	70%*
	14	Hospital admin EHR tasks: h + p update, admit order, OR orders	75%*
Initial Operating Room Visit	15	Set room temp 69F/21C (AORN)	60%
	16	Review surgical plan with staff and positioning	94%**
	17	Welcome and Remind Anesthesia/CRNA: TXA; Sufenta instead of Remi; Avoid Ketamine, MAP & BIS Goals	78%*
	18	Anesthesia: airway concerns?	79%*
	19	Room Equip: fluid warmer, 2 lightboxes, lat XR holder, lead shield	55%
	20	Confirm cell saver ordered and machine in room	67%
	21	Confirm imaging ordered including large field C-arm flouro	69%
	22	Instrument layout photograph available	20%
	23	Specials for today. All equipment available?	75%*
	24	Give circulator saline: betadine concentration 3:1	22%
	25	Check Jackson Table pin position, and test up/down/tberg/locking	68%
	26	Thigh foam pad and 3 pillows at foot to keep knee caps free	72%*
	27	Stool under table for X-ray	14%
	28	Put on under-body warmer on Jackson Table; connect and turn on warmers × 2	71%*
	29	Sutures for the Field: VLock 3–0 × 2, Quill or StrataFix #2 double-ended	30%
	30	Bone graft ordered	53%
	31	Dural repair supplies in room: 6–0 Proline, Duraseal, Surgicel	45%
	32	Diamond and metal cutting bur available in room	45%
	33	Prep table setup: sterile 4 × 4’s, alcohol, ChloroPrep × 2, sterile pen, Integuseal	47%
	34	Reminder: keep patient warm	67%
	35	Answer any questions that they have from OR team. Thank You for Help	75%*
Before Turning	36	In OR time (wheels in)	69%
	37	Tape up all preop imaging on the wall, and/or pull up digitally on monitor	72%*
	38	Surgical implant “map” and other diagrams posted	53%
	39	Confirm vertebral numbering/transitional anatomy	72%*
	40	Put warm blankets on patient immediately	67%
	41	Double-check Jackson Table pad positions for patient size	79%*
	42	IV’s flowing normally, caps tight, no kinks, taped secure, no pressure from shutoff valves	72%*
	43	Foley placed with good urine flow	76%**
	44	Pneumatic boots applied (non-pediatric) and turned on	67%
	45	Evoked potential wire placement done	81%*
	46	BIS monitor on forehead	65%
	47	Endo-tracheal tube taped securely & tube depth (cm)—confirmed 2 team memb	82%**
	48	Bite block × 2 between molars, w test jaw close, confirmed 2 team memb, double-check	72%**
	49	Stretcher/Jackson Table heights set correctly and tables locked	69%
	50	Ensure all lines, Foley, wires are free, Foley threaded through under-warmer	71%*
	51	NM Tech: identify needle locations for staff safety and wrists/hands wrapped w towel	63%
	52	Confirm grounding pad not over needles	75%*
Positioning	53	Patient gently turned to prone position with my help, arms positioned	72%*
	54	Face carefully positioned in prone view with eyes protected and visible w/o pressure (2 team memb cross-check)	71%**
	55	Reverse Trendelenberg approx 4 degrees—eye/airway protection (Lenke)	60%
	56	Check that all IV and arterial connectors all double-checked tight	80%*
	57	ET tube and bite blocks secure and proper depth, tight connections (2 team member cross-check)	79%**
	58	Lips and ears without pressure (2 team memb cross-check)	78%*
	59	Chest roll is away from the brachial plexus and airway (2 team memb)	78%*
	60	IV’s, arterial lines flowing, no infiltrates, no kinks, caps tight, no skin pressure from valves, etc. (comp syndrome)	72%*
	61	Neck in neutral position (2 team memb)	78%**
	62	For women, adjust breasts to minimize pressure	79%*
	63	New warm blankets on entire patient	44%
	64	Turn on warmers, on high	72%*
	65	Elbows and shoulders at 90 degrees, no pressure ulnar nerve	78%**
	66	Arm boards out of way for surgeon, all connectors tight	76%*
	67	Pneumatic boots on, plugged in and turned on	67%
	68	Add additional foam under iliac wings if needed for thin patients	72%*
	69	Pelvis level, thighs on pads, knees no pressure, legs on pillows w knees flexed, strap on	69%
	70	Under PT: abdomen, G-Tubes, ITB pumps hanging free without pressure	65%
	71	Under PT: genitals free of pressure, not lying on Foley valve	76%**
	72	Under patient: Tape/Velcro up Foley and NM wires to be out of way of C-arm	71%*
Prep and Drape	73	If needed, shoulders/buttock taped to prevent creasing	47%
	74	Set tracker/timers for OR time, surgical time, q30m anesth and leg check, 90 m family call, 2 h glove change	65%
	75	Upper and lower warm blankets applied	56%
	76	4 × 4 sterile sponge alcohol wipe down entire back, avoiding pooling for fire risk avoidance (SRS 2017)	65%
	77	Surgical lights lined up over the surgical field, “kissing”, high enough to avoid head bump	68%
	78	Stick-on plastic “1010” drapes at least 3 inches from expected incision ends, 5 + inches each side	47%
	79	Tape top 1010 up to blankets to prevent contamination	30%
	80	Chlorohexidine dry x3min and no “pooling” on field or on sheets below (fire risk)	72%*
	81	Skin incision and cross-hatches marked sterile marker	61%
	82	Large sticky drape, splits, and edges sealed with sticky drapes	68%
	83	Red banner or tape across main OR door (infection prev)	61%
	84	Confirm patient stretcher location outside door with room label (emergent flip)	70%*
	85	Trip hazard removal and check: tape down or remove all wires/tubes on floor	59%
	86	Family phone number and name on white board	43%
	87	Set Bovie 50coag, 70cut, Bip 70, TPS STRYKER NEURO Pi DRILL ACCELERATION & ORQUE 100%	33%
Pre-Incision Timeout	88	SURGEON: welcome and team introductions name and role, names on white board	71%*
	89	SURGEON: briefing begins: patient/family intro and surgical plan, EBL	88%*
	90	SURGEON: reviews preop imaging: confirmed patient ID, levels, transitional anatomy?	81%*
	91	Circ RN: patient name, DOB confirmed with anesthesia	88%*
	92	Circ RN: surgery consent read aloud	93%*
	93	Circ RN: allergies/latex allergies	87%**
	94	Circ RN: positioning confirm legs/feet ok and strap on legs	71%*
	95	ANESTH: antibiotic given within 1 h skin incision	93%**
	96	ANESTH: positioning confirm OK eyes, nose, mouth, bite block, ears, neck, arms/shoulders	76%*
	97	ANESTH: warm all fluids, keep warmers on high until patient 37c	67%
	98	ANESTH: minimize fresh gas flow to move bellows (1 L)—optimize body temp	55%
	99	ANESTH: TXA given and drip started (50 mg/kg, 5 mg/kg/h); run until PACU	64%
	100	ANESTH: MAP in 70’s exposure, 80’s during rod insertion	67%
	101	ANESTH: verbally report eyes, ET tube and hands OK; EBL,MAP,PPV,BIS,TEMP,UO q30	61%
	102	Circ RN: IF ARMS TUCKED, verbal “Hands OK” q30m	53%
	103	Circ RN: verbal “Legs OK” q30m	53%
	104	Timers set for q30 min Anesth and foot/leg checks, 90 m fam call, 2 h glove changes	52%
	105	ASSISTANT: spinal “RED ZONE” education L1 and above	45%
	106	NEUROMONITORING: being performed and baseline readings	93%**
	107	Neuromonitoring change emergency protocol review—Circ Nurse will run, copies posted and available	83%*
	108	Fire safety review: no alcohol/CHG pooling. Prep dry. Emergency checklist available	56%
	109	Malignant hyperthermia risk/cart & dantrolene location	67%
	110	Safety zone and sharps awareness—pass blunt end first to table	50%
	111	Keep light handles 4in/10 cm above highest head (infection prev)	42%
	112	Confirm floor clear of trip hazards, all cables/tubes taped down	47%
	113	Verify bed location and label in case of emergency flip needed	50%
	114	Make sure everyone is double gloved	50%
	115	Minimize trips going in and out of room unless necessary	68%
	116	Sit down immediately if light-headed and notify staff (falls prevention)	42%
	117	Keep talking to minimum to maximize communication, minimize distraction	40%
	118	Use verbal “readbacks” to ensure communication completed accurately	68%
	119	Any other team concerns?	76%
	120	Encourage all team members to speak up if there is concern or question	78%*
	121	All team members agree with completed timeout and reply with “Aye”	61%**
Intraoperative	122	Skin incised lightly. Surgical start time	53%
	123	Circulating nurse calls family for first phone call, marked on board	62%
	124	Penfield 4 over transverse process for level confirmation, skin marked	45%
	125	Take off the sucker-tip to avoid betadine in cell saver (Dan Woodfin)	27%
	126	Incision covered with green towel and sponge placed in wound	40%
	127	Help X-ray Tech align C-arm or X-ray to decrease X-ray exposure	58%
	128	AP/Lat flouroscopy/X-ray: confirm Penfield position above sacrum, confirmed 3 team members	63%
	129	Facet joint and lamina marked with bur adjacent to Penfield 4	36%
	130	For short incisions, pass pedicle probe part-way down pedicle and reshoot flouro/X-ray to confirm	50%
	131	Marked level written on white board	32%
	132	Hemostasis check completed left side (every 10 cm during exposure each side)	33%
	133	Vancomycin powder 1gm rubbed into muscle left side (Lenke Oct 2016)	35%
	134	Hemostasis check completed right side	29%
	135	Vancomycin powder 1gm rubbed into muscle right side	35%
	136	Exposure complete time	41%
	137	Screw implant insertion begin time	45%
	138	Pedicle screw placement safety subroutine (repeat)	56%
	139	Each step called out verbally as completed to NM and checklist team members	35%
Finishing Screws and Confirming Imaging	140	All pedicle screws left side completed	67%
	141	All pedicle screws right side completed	65%
	142	Bilateral iliac wing screws completed, if needed	59%
	143	Dilute betadine solution and sponge in wound, sterile towel cover w marks	10%
	144	Flouroscopic/X-ray confirmation screw position	75%*
Final Rods and Locking	145	Rods inserted bilaterally with multiple persuaders, compression and distraction	59%
	146	Time spine implants completed	53%
	147	Ensure there is adequate rod sticking out each end, no muscle entrapment	61%
	148	Dilute betadine placed in wound, incision covered and marked	20%
	149	X-rays: good coronal/sagittal balance, rod lengths	53%
Prior to closure	150	Screw caps double checked by surgeon and assistant with verbal check each cap. (Vitale M-2016 Pt Safety Conf)	75%*
	151	Valsalva maneuver done to confirm no spinal fluid leak	53%
	152	Irrigate wound copiously with saline	75%*
	153	Complete posterolateral decortication, and place bone graft mixed w 2 g vancomycin	75%*
	154	Meticulous hemostasis confirmed by careful wound inspection top to bottom bilat	59%
	155	Drain placement if needed	59%
	156	Blood loss/added	65%
Closure	157	Note closure begin time	52%
	158	Fascia closure at distal and proximal ends first, then middle	37%
	159	Great care to ensure drain not sutured in, deep and superficial	63%
	160	Push down on wound to test for water-tight closure	42%
	161	Running #1 Vicryl or #2 Quill on fascia to further water-seal fascia	53%
	162	Confirm body weight, then inject 0.5% Marcaine with epi up to 30 cc if wt > = 60 kg	21%
	163	Incision closed time. (surgery end)	53%
	164	Dermabond and Steri-Strips applied and allowed to dry on incision	31%
	165	Confirm evoked potential and emg monitoring was normal throughout surgery	75%*
	166	Remove ALL non-disposables from drapes (bipolar, clips, cables, etc.)	59%
	167	Pre-turn check for all lines/Foley. Loosen Foley from table	63%
Turn to supine	168	Turn supine onto bed gently	59%
	169	Inspect face, arms, hands, chest, pelvis and lower extremity for skin/pressure/swelling	63%
	170	Patient opens eyes	53%
	171	Extubated time	59%
	172	Tongue check: no laceration	65%
	173	All neuromonitoring needles removed and accounted for. (ELI Nov 2017)	75%*
	174	Patient moved bilateral lower extremities	81%**
	175	Extubated and moving lower extremities (wakeup done)	75%*
	176	Time patient leaves room (wheels out)	65%
	177	Complete operative note with merged eChecklist log, pasting into Hosp EHR	53%
	178	Ask for family to be put into consult room	56%
Checkout/Debriefing	179	What went well in the surgery?	76%*
	180	Complications?	83%*
	181	Any equipment problems? (blank = no)	78%*
	182	Any preventable trips in/out of OR room?	67%
	183	Anything we could have done to make safer/more efficient? (ELI’s—error/event, learn improve) (blank = no)	76%*
	184	Suggested changes to checklists?	71%*
	185	Thank everyone for their help, recognizing them for things done well	80%*
	186	Sustainability/stewardship waste avoidance	65%
	187	Any new “Pearls” teaching points?	65%

** ≥ 70% of respondents selected “very important, must be included”; * ≥ 70% of respondents selected “moderately important” or “very important”

Items that are more common/unique in spinal deformity surgery include prone and lateral positioning, neuromonitoring baseline readings, double-checking that the implant screw caps are locked prior to closure, and confirming that the patient was moving bilateral lower extremities before leaving the operating room when possible. The final checklist adopted and now distributed by SRS is shown in Supplementary File 1.

## Discussion

Ensuring safety in spinal deformity surgery is of paramount importance. This study, a survey of experienced scoliosis surgeons, identified 74 items considered important to include in a perioperative checklist specific for spinal deformity. Seventeen of these items were considered “very important” to include by more than 70% of respondents. This study is the first to provide a comprehensive perioperative spinal deformity surgery safety checklist, for the whole surgical episode from preoperative holding to discharge from the operating room. Importantly, three of the sections with the most items considered important for publication were actions that can be performed before (Pre-Operative Area and Positioning) and after surgery (Checkout/Debriefing). This underscores the perceived need by the panelists for well-designed and checked processes in the operating room environment, not just during the surgical/technical procedure itself. The proposed checklist is not meant to lead to duplication of existing hospital/surgeon checklists, but rather stimulate spinal deformity surgeons and teams to adapt to their local institutional setting.

### Interpretation and generalizability

**Fig. 2:**
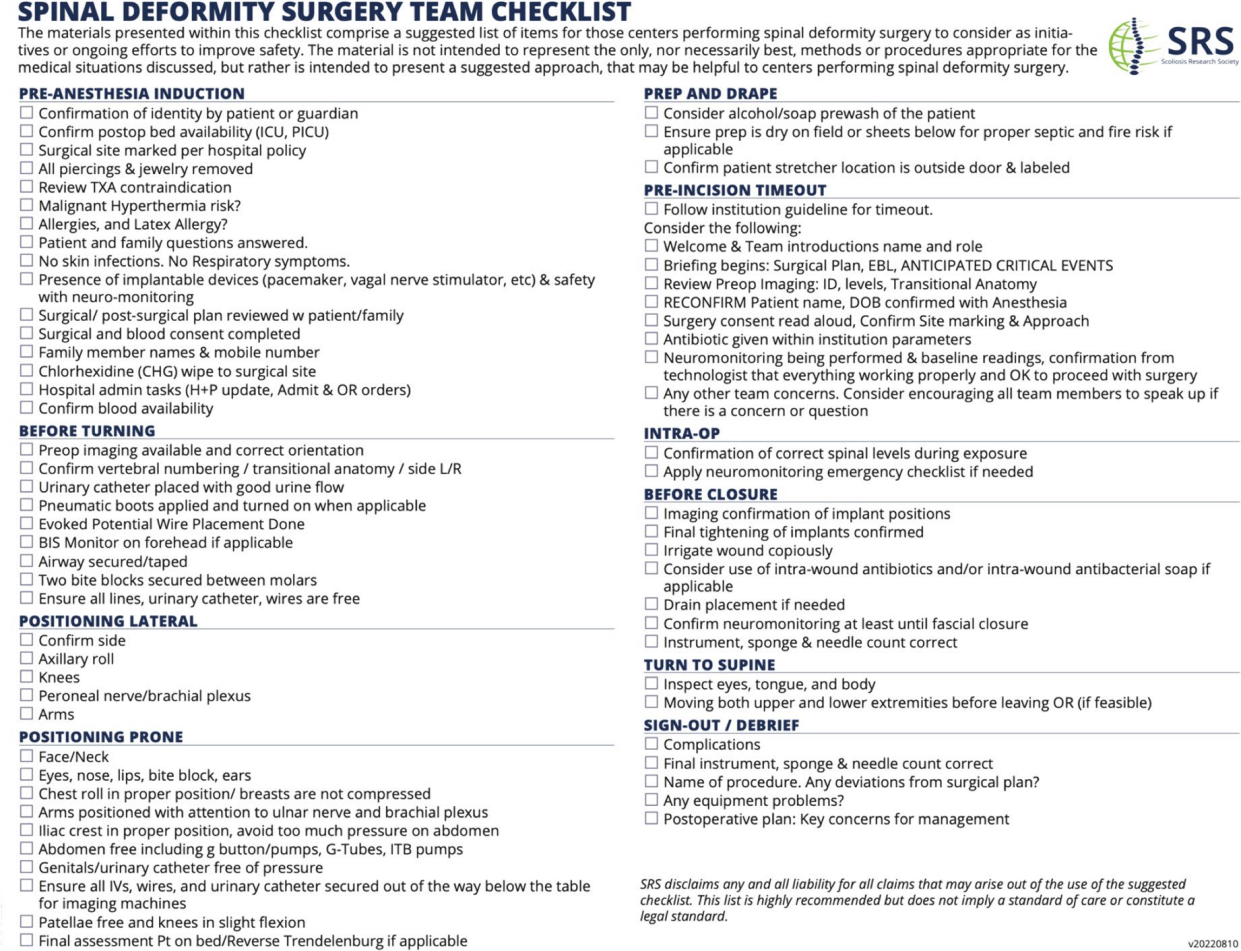
Final SRS spinal deformity surgery team checklist

The implementation of checklists has improved surgical safety worldwide [6, 8]. These checklists ensure everyone involved in patient care is aware of the actions that need to be taken to ensure safety and promotes collaboration across providers, increased awareness, and effective communication. Recently, the need for team checklists has become more important since the increase in health care professional turnover in operating rooms related to the COVID-19 pandemic, frequently resulting in temporary operating room nurses, scrub technicians, and anesthesia staff being part of the surgical team who are less familiar with standard operating practices (Fig. 2).

In degenerative spine surgery, Kulkarni et al. developed a checklist with an aim of reducing human error and major perioperative complications [13]. A total of 858 spine surgery patients were divided into two groups (before and after implementation of the checklist). This checklist consisted of 16 preoperative items (including patient data, anticipation of intensive care unit admission, planned procedure, and planned use of implants/grafts), 13 intraoperative items (including use of antibiotics, adequate patient positioning, hemostasis, and adequate gauze/Gamzee counts), and 8 postoperative items (including vital sign checks, neurological checks, and physiotherapy plan) [13]. Authors reported a significant reduction in preventable adverse events from 14.2% to 3.5% after the checklist was implemented, highlighting the potential benefit of this strategy [13].

Complication rates in spinal deformity surgery are high, especially in the ASD population. A prospective multi-center study of 291 patients revealed 52.5% of patients were affected by at least one perioperative complication [14]. These included implant-related complications (27.8%), neurological (27.8%), infectious (14.8%), and wound complications (5.2%), among others [14]. Preventable complications also occur in the pediatric and adolescent patient spinal deformity surgical populations, in critical areas such as positioning, wound infection, implant failures, and new neurologic deficits. While strategies such as hypotension avoidance, use of incentive spirometry, use of neuromonitoring, and use of prophylactic anticoagulation can reduce the risk of medical complications [15], team efforts that are a fundamental part of safe spinal deformity surgery can be enabled by implementing standard operating procedures and safety checklists such as the one we have designed. For example, Sethi et al. described The Seattle Spine Team Protocol, a systematic multidisciplinary effort consisting of multidisciplinary spine conferences, a mandatory patient education course, a dual-attending-surgeon approach, a dedicated specialist anesthesia team, and rigorous intraoperative monitoring of blood loss and coagulopathy [16]. Authors compared outcomes of patients treated before and after implementation of this protocol, finding that the latter half of patients had significant reductions across all complications including cardiovascular events, infections, and implant failures [15].

Perhaps one of the pinnacles of safety in spinal deformity was the development of a checklist to respond to intraoperative changes in neuromonitoring [10]. Though rare, neurologic complications are some of the most feared and also potentially devastating adverse events. Through a Delphi approach, a team of 21 spinal deformity experts and 1 neurologist developed a checklist to promote a team approach to changes in neuromonitoring and, thus, decrease variability in neuromonitoring practices [10] Similar to that checklist, our objective was to develop a list to improve safety in spinal deformity surgery, which will need to be validated in future investigations. Likewise, the utility of modified checklists based on input from other operating room staff and adaptations at different institutions will need to be validated.

### Limitations

The first limitation of this study is that only surgeon stakeholders were assessed, and no other key stakeholders such as patients and family members, or other operating room team members. The sample size of the survey may also be a limitation, as 25 surgeons (70%) completed the survey in its entirety. The choice of the 70% cutoff for item inclusion may also be considered a limitation since some items came close to that cutoff and were not included. Surgeons and their teams are encouraged to review the full list of original checklist items as a potential source of items to include in their own lists. The aim of the current study was to create a concise checklist, granular enough to include the important items, yet not so detailed that it would be cumbersome to implement. As a result, another limitation of this study is that there was no available objective data on the number of items in a checklist that can be deemed optimal and acceptable in an operating room environment during multi-hour surgical procedures, and therefore we do not know if the right balance was found. Further work will need to study this continuous cost–benefit balancing. Potential time savings from using the checklist, in terms of improved wakeup times, less staff running for missing equipment, etc., was also not examined in this study.

## Conclusion

Current surgery checklists have not been adapted to the specific needs of spine deformity surgery. This study is the first to provide a comprehensive perioperative spinal deformity surgery safety checklist, for the whole surgical episode from preoperative holding to discharge from the operating room. Many items of the checklist are unique to spine deformity surgery. We encourage surgical teams across the world to implement it in their own health care environment and adapt as needed to the local environment. Further studies are needed to study the effectiveness of implementing surgical checklists, and how to best encourage their use routinely in spinal deformity and other surgeries.

## Supplementary Information

Below is the link to the electronic supplementary material.

Supplementary file1 (PDF 111 kb)

## Data Availability

Yes.
