# Supplementary material for: Development of the scoliosis research society spinal deformity surgery safety checklist
Source: Spine Deform. 2024 Oct 23;13(2):327–38. doi: 10.1007/s43390-024-00973-1 (PMC11893681; doi:10.1007/s43390-024-00973-1)
Supplement: Supplementary file 1 — Supplementary file1 (PDF 111 kb) [file 43390_2024_973_MOESM1_ESM.pdf]

SPINAL DEFORMITY SURGERY TEAM CHECKLIST

SRS Scoliosis Research Society

The materials presented within this checklist comprise a suggested list of items for those centers performing spinal deformity surgery to consider as initiatives or ongoing efforts to improve safety. The material is not intended to represent the only, nor necessarily best, methods or procedures appropriate for the medical situations discussed, but rather is intended to present a suggested approach, that may be helpful to centers performing spinal deformity surgery.

PRE-ANESTHESIA INDUCTION

- ☐ Confirmation of identity by patient or guardian
- ☐ Confirm postop bed availability (ICU, PICU)
- ☐ Surgical site marked per hospital policy
- ☐ All piercings & jewelry removed
- ☐ Review TXA contraindication
- ☐ Malignant Hyperthermia risk?
- ☐ Allergies, and Latex Allergy?
- ☐ Patient and family questions answered.
- ☐ No skin infections. No Respiratory symptoms.
- ☐ Presence of implantable devices (pacemaker, vagal nerve stimulator, etc) & safety with neuro-monitoring
- ☐ Surgical/ post-surgical plan reviewed w patient/family
- ☐ Surgical and blood consent completed
- ☐ Family member names & mobile number
- ☐ Chlorhexidine (CHG) wipe to surgical site
- ☐ Hospital admin tasks (H+P update, Admit & OR orders)
- ☐ Confirm blood availability

BEFORE TURNING

- ☐ Preop imaging available and correct orientation
- ☐ Confirm vertebral numbering / transitional anatomy / side L/R
- ☐ Urinary catheter placed with good urine flow
- ☐ Pneumatic boots applied and turned on when applicable
- ☐ Evoked Potential Wire Placement Done
- ☐ BIS Monitor on forehead if applicable
- ☐ Airway secured/taped
- ☐ Two bite blocks secured between molars
- ☐ Ensure all lines, urinary catheter, wires are free

POSITIONING LATERAL

- ☐ Confirm side
- ☐ Axillary roll
- ☐ Knees
- ☐ Peroneal nerve/brachial plexus
- ☐ Arms

POSITIONING PRONE

- ☐ Face/Neck
- ☐ Eyes, nose, lips, bite block, ears
- ☐ Chest roll in proper position/ breasts are not compressed
- ☐ Arms positioned with attention to ulnar nerve and brachial plexus
- ☐ Iliac crest in proper position, avoid too much pressure on abdomen
- ☐ Abdomen free including g button/pumps, G-Tubes, ITB pumps
- ☐ Genitals/urinary catheter free of pressure
- ☐ Ensure all IVs, wires, and urinary catheter secured out of the way below the table for imaging machines
- ☐ Patellae free and knees in slight flexion
- ☐ Final assessment Pt on bed/Reverse Trendelenburg if applicable

PREP AND DRAPE

- ☐ Consider alcohol/soap prewash of the patient
- ☐ Ensure prep is dry on field or sheets below for proper septic and fire risk if applicable
- ☐ Confirm patient stretcher location is outside door & labeled

PRE-INCISION TIMEOUT

- ☐ Follow institution guideline for timeout.

Consider the following:

- ☐ Welcome & Team introductions name and role
- ☐ Briefing begins: Surgical Plan, EBL, ANTICIPATED CRITICAL EVENTS
- ☐ Review Preop Imaging: ID, levels, Transitional Anatomy
- ☐ RECONFIRM Patient name, DOB confirmed with Anesthesia
- ☐ Surgery consent read aloud, Confirm Site marking & Approach
- ☐ Antibiotic given within institution parameters
- ☐ Neuromonitoring being performed & baseline readings, confirmation from technologist that everything working properly and OK to proceed with surgery
- ☐ Any other team concerns. Consider encouraging all team members to speak up if there is a concern or question

INTRA-OP

- ☐ Confirmation of correct spinal levels during exposure
- ☐ Apply neuromonitoring emergency checklist if needed

BEFORE CLOSURE

- ☐ Imaging confirmation of implant positions
- ☐ Final tightening of implants confirmed
- ☐ Irrigate wound copiously
- ☐ Consider use of intra-wound antibiotics and/or intra-wound antibacterial soap if applicable
- ☐ Drain placement if needed
- ☐ Confirm neuromonitoring at least until fascial closure
- ☐ Instrument, sponge & needle count correct

TURN TO SUPINE

- ☐ Inspect eyes, tongue, and body
- ☐ Moving both upper and lower extremities before leaving OR (if feasible)

SIGN-OUT / DEBRIEF

- ☐ Complications
- ☐ Final instrument, sponge & needle count correct
- ☐ Name of procedure. Any deviations from surgical plan?
- ☐ Any equipment problems?
- ☐ Postoperative plan: Key concerns for management

SRS disclaims any and all liability for all claims that may arise out of the use of the suggested checklist. This list is highly recommended but does not imply a standard of care or constitute a legal standard.

v20220810

SRS-0822-144
